# GrimACE: automated, multimodal cage-side assessment of pain and well-being in mice

**DOI:** 10.1038/s41684-026-01695-9

**Published:** 2026-03-05

**Authors:** Oliver Sturman, Marcel Schmutz, Tom Lorimer, Runzhong Zhang, Mattia Privitera, Fabienne K. Roessler, Justine Leonardi, Rebecca Waag, Alina-Mariuca Marinescu, Clara Bekemeier, Katharina Hohlbaum, Johannes Bohacek

**Affiliations:** 1 Laboratory of Molecular and Behavioral Neuroscience, Institute for Neuroscience, Department of Health Sciences and Technology, ETH, Zurich, Switzerland; 2 Neuroscience Center Zurich, ETH Zurich and University of Zurich, Zurich, Switzerland; 3 ETH Zurich 3R Hub, ETH, Zurich, Switzerland; 4 Institute of Animal Welfare Animal Behavior and Laboratory Animal Science School of Veterinary Medicine Freie Universitat Berlin, Berlin, Germany; 5 Science of Intelligence, Research Cluster of Excellence, Berlin, Germany; 6 German Centre for the Protection of Laboratory Animals (Bf3R) German Federal Institute for Risk Assessment (BfR), Berlin, Germany

**Keywords:** Neuroscience, Animal behaviour, Animal physiology

## Abstract

Pain and welfare monitoring is essential for ethical animal testing, but current cage-side assessments are qualitative and subjective. Here we present the GrimACE, a fully standardized and automated cage-side monitoring tool for mice, the most widely used animals in research. The GrimACE uses computer vision to provide automated mouse grimace scale (MGS) assessment together with pose estimation in a safe, dark environment. We validated the system by analyzing pain after brain surgeries (craniotomies) with head implants under two analgesia regimes. Human-expert and automated MGS scores showed very high correlation (Pearson’s *r* = 0.87). Both expert and automated scores revealed that a moderate increase in pain can be detected for up to 48 h after surgeries, but that both a single dose of meloxicam (5 mg/kg subcutaneuously) or three doses of buprenorphine (0.1 mg/kg) + meloxicam (5 mg/kg subcutaneuously) provide adequate and comparable pain management. Simultaneous pose estimation demonstrated that mice receiving buprenorphine + meloxicam showed increased movement 4 h after surgery, indicative of hyperactivity, a well-known side effect of opioid treatment. Significant weight loss was also detected in the buprenorphine + meloxicam treatment group compared with the meloxicam-only group. In addition, detailed BehaviorFlow analysis and automated MGS scoring of control animals suggests that habituation to GrimACE is unnecessary, and that measurements can be repeated multiple times, ensuring standardized postoperative recovery monitoring.

## Abstract (web summary)

The authors present the GrimACE, an automated cage-side system that uses computer vision to score mouse pain and behavior. The tool, which detects postsurgery pain and analgesic effects, enables repeated, standardized welfare monitoring.

## Main

The evaluation of pain and well-being in laboratory animals is an essential part of all ethical experimentation^1,2^. Although mice are the most commonly used animal model in scientific studies owing to their genetic similarities to humans and their utility in understanding various diseases and treatments^3,4^, accurate assessment of pain and well-being in mice is often challenging. Inadequate pain management not only raises serious ethical concerns regarding the humane treatment of animals, but also jeopardizes the validity and reproducibility of research findings^5,6^. With good pain and welfare monitoring protocols in place, it is possible to design appropriate analgesia regimes, detect problems before they become too severe, and define and work with humane endpoints.

Moderate-to-severe pain in laboratory mice is most often related to surgical interventions, which are a cornerstone of in vivo animal research. The gold standard to assess postsurgical recovery is based on cage-side assessment^7^, where key behavioral parameters (for example, posture, coat condition, movement patterns and wound licking) are assessed by a trained experimenter via visual inspection^2,8–10^. These visual observations, which provide qualitative scores or counts for individual parameters, are well suited for rapid assessment of postsurgical recovery by personnel trained in animal experimentation or by trained animal caretakers. However, cage-side assessment is prone to bias, subjectivity and poor sensitivity to subtle alterations in well-being^11^. It is also widely believed that prey animals may hide signs of pain, making it difficult for observers in close proximity to accurately assess subtle changes in well-being^12^. By contrast, measures such as telemetry for movement and heart rate, nest-building behavior or burrowing behavior have been shown to be more sensitive indicators of postsurgical pain and recovery, as they can reveal pain-related changes when standard cage-side assessment fails to reveal impairments^13–17^. However, these tests initially require surgeries to implant transmitters or habituation and then prolonged observation periods in single-housing conditions to analyze complex behaviors, rendering these tests impractical for routine use in laboratories that do not specialize in pain assessment. Over the past decade, the assessment of facial features to detect the affective component of pain has been popularized through the development of the mouse grimace scale (MGS)^18,19^. This approach requires minimal habituation and only brief periods of surveillance using photo or video recordings. Subsequent manual scoring assesses whether signs of pain can be detected across five facial features (orbital tightening, nose bulge, cheek bulge, ear position and whisker change), and each feature is assigned a value from 0 to 2 (0 = absent, 1 = moderate, 2 = severe). This scoring process is very labor intensive, requires highly trained experimenters^18,20^ and remains subject to bias^21^. Several groups have developed pipelines to automate (parts of) this process^22–26^; however, since experimental setups vary between labs, automated pipelines do not transfer well between labs and setups. Moreover, in many scenarios, the assessment of grimace scores with automated software is particularly challenging, such as when head implants or other interventions (fresh wound sites on the head with ointment from sterilization and local anesthetics) alter the images. This is particularly problematic, as craniotomies are the most commonly used surgical procedures in neuroscience research, and pain associated with craniotomies is notoriously difficult to detect using cage-side assessment^27,28^. MGS scores are highly sensitive to pain after craniotomies, showing that pain typically peaks 4–6 h after surgery, before resolving gradually over the course of 24–48 h (refs. ^6,9,27,29^). These studies also suggest that nonsteroidal anti-inflammatory drugs (NSAIDs) such as meloxicam or carprofen provide adequate analgesia^27,29^. Finally, going beyond classical pain assessment tools, deep behavioral profiling has recently emerged for pain detection, leveraging machine learning to extract subtle behavioral motifs from video recordings of freely moving animals^30–32^. These approaches were able to reveal subtle behavioral alterations in mouse pain models^30^, resolve a broad range of effects following stress exposure^33^ and distinguish the impact of different pharmacological interventions^34^. However, these tools are still difficult to implement in most labs, as they require substantial technical expertise^35^.

Here we present the GrimACE, a robust and portable cage-side assessment system that minimizes stress levels (safe, dark environment), collects high-quality videos with full-body pose estimation for behavioral profiling and acquires standardized high-quality facial images with automated MGS scoring trained on data from expert human raters. We applied deep behavioral phenotyping on the pose estimation data using our newly developed BehaviorFlow pipeline^33^. We tested the GrimACE device by assessing postoperative analgesia following craniotomies with different types of brain implant, comparing NSAID with opioid + NSAID analgesia. We show that, from 10-min recordings, the GrimACE can automatically detect mild-to-moderate postoperative pain from facial features, when cage-side assessment does not. Behavioral profiling readily revealed known side effects of opioid treatment and together with the MGS allowed the tracking of recovery over time.

## Results

### The GrimACE

**Fig. 1:**
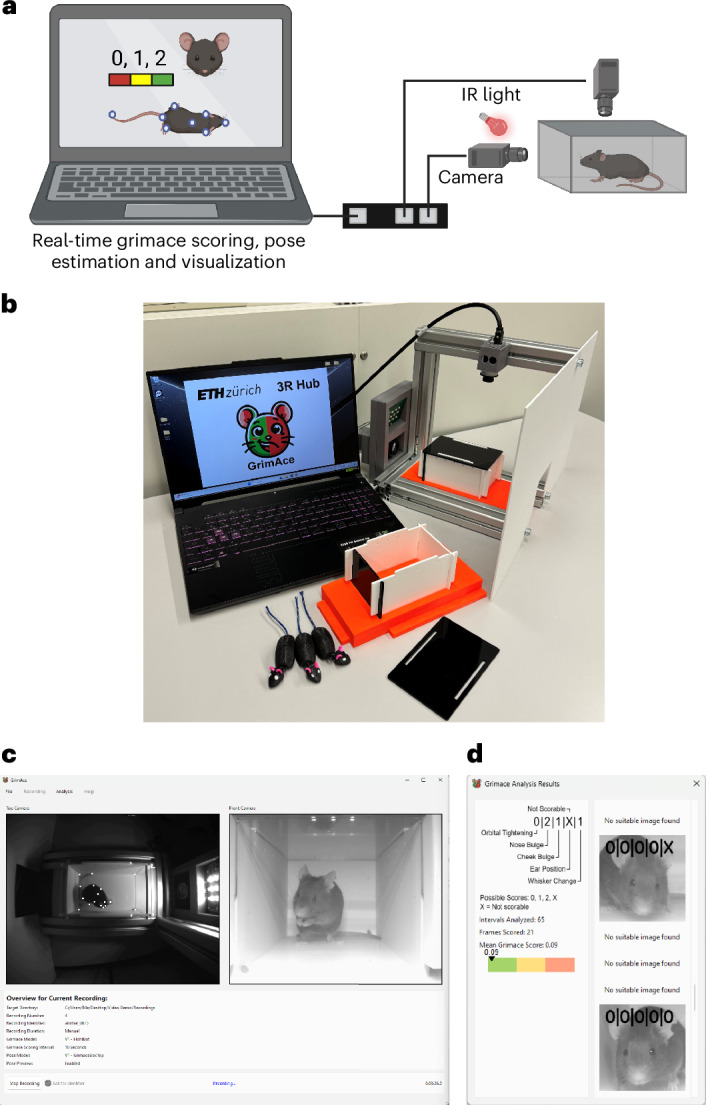
The GrimACE, a complete hardware and software solution for standardized MGS scoring and key point-based pose estimation. **a**, Schematic of the GrimACE. **b**, Picture of the GrimACE, with extra arena and 3D-printed mice that can be used for demonstrative purposes (top acrylic shield has been removed to increase visibility for this photograph). **c**, The GrimACE app during a recording, with the pose-estimation video on the left and MGS video on the right. **d**, The live MGS window, with scored images, the current mean score and detailed score breakdowns. A video of the software running is available via Zenodo at https://zenodo.org/records/15119195. Icons created in BioRender; Bohacek, J. https://biorender.com/oey5tf6 (2026).

The GrimACE is a complete hardware and software solution for standardized MGS image acquisition, scoring and key point-based pose estimation. The GrimACE consists of an aluminum frame with custom three-dimensionally (3D)-printed (polylactic acid, PLA) components to hold lights, a front and top camera and an acrylic arena with similar dimensions to other MGS arenas^24,29,36,37^ (Fig. 1a,b). The arena has matte white walls and an infrared (IR)-permeable front and lid. The use of IR-permeable black acrylic on the front and lid of the box allows a clear view of the mouse from the front and above, creates a calming dark environment for the mouse and ensures that the lighting of the room has minimal effect on the image/video quality. The arena also has small air holes to ensure that the mouse remains comfortable. The arena is mounted on an orange 3D-printed base that slides into a standardized position within the frame, ensuring repeatability. The matte white acrylic and orange PLA help to reduce reflections and provide good image contrast. Large matte white acrylic sheets attached to the aluminum frame help to reduce reflections from external light sources. The small acrylic box and 3D-printed base have also been designed to fit together without glue or screws so that they can be dismantled and reassembled in seconds by hand, which makes them easy to clean. Once the mouse is in the arena and is in position, the user can trigger the system to begin recording video, acquiring and analyzing images using the GrimACE app.

### The GrimACE app and machine learning algorithm overview

The GrimACE app provides a clean, simple, easy-to-use interface for running MGS scoring and pose estimation. The user sets the output file naming convention (for example, an experiment name prefix), the desired test duration (for example, 10 min) and the MGS scoring interval (for example, 10 s). These settings persist from test to test. When the mouse is in position, the app is triggered manually and displays several real-time quality checks throughout the test: front video, top video (including pose estimation) (Fig. 1c) and cropped scored images with detailed score breakdowns (Fig. 1d). An example video demonstrating the real-time implementation of the GrimACE app is available via Zenodo at 10.5281/zenodo.15119195. At the end of each test, two videos (top and front) and three csv files are saved (pose estimation, grimace scores with corresponding video frame numbers and all video frame timestamps). The pose estimation data are directly compatible with state-of-the-art behavioral analysis pipelines and techniques such as BehaviorFlow^33,38^.

Behind the scenes, the machine learning models perform two main tasks: pose estimation (top camera) and MGS image scoring (front camera). Pose estimation is performed using YOLO^39^ (see Methods for details), while MGS image scoring is split into three tasks: identifying high-quality frames suitable for MGS scoring; detecting the position of the mouse’s face; and determining the MGS score. The MGS scoring tasks are performed by three neural networks in series. The frame quality network, trained on 3,200 manually annotated images and based on a modified mobilenetv3 network, scores each video frame on a continuous scale from 0 to 6 based on image quality, head position and motion blur to find the best frames for MGS scoring. The mouse face detection network, trained on 1,076 manually labeled images and based on YOLOv8s^39,40^, draws a bounding box around the mouse face and passes the cropped image to the MGS score network. The MGS score network, trained on 1,245 expert-scored images and based on a modified PyTorch Visiontransformer vit_b_16^41,42^ connected to two ReLU hidden layers and 5 linear activation cross-entropy output heads, gives a score (0, 1, 2 or ‘not rateable’) for each of the 5 facial features (orbital tightening, nose bulge, cheek bulge, ear position and whisker change). In all results reported here, multifold cross-validation was used to ensure that the model scoring each mouse had not been trained on any data from that mouse in any of the networks. For full network and training details, see Methods.

### Initial validation and testing of the GrimACE (experiment 1)

**Fig. 2:**
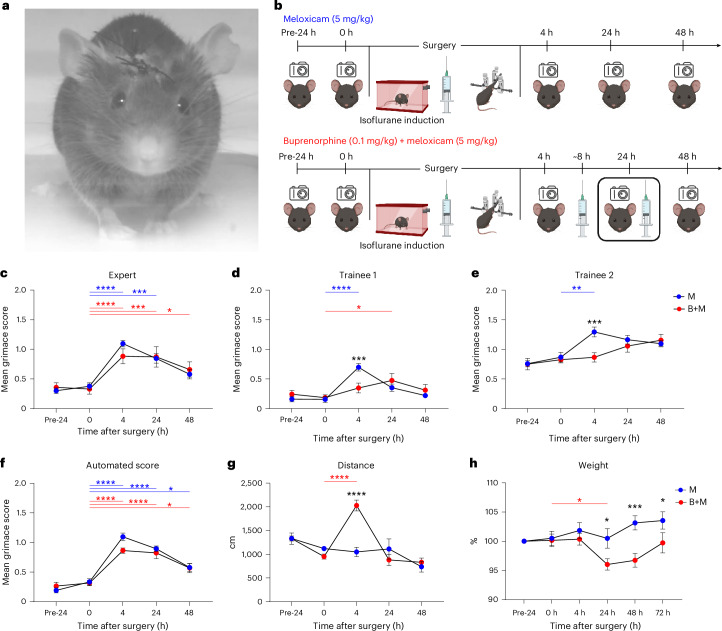
GrimACE assessment after craniotomy with fiberoptic implants. **a**, An animal 24 h after surgery. **b**, Experimental timeline showing GrimACE recordings, surgery and injections of analgesia (meloxicam: 8 mice; meloxicam + buprenorphine: 7 mice). **c**, MGS scores from the expert rater show a significant main effect of time (*F*(4,50) = 31.37, *P* ≤ 0.0001), but no effect of treatment and no time × treatment interaction. **d**, MGS scores from trainee 1 show a significant main effect of time (*F*(4,50) = 11.41, *P* < 0.0001), no effect of treatment, and a significant time × treatment interaction (*F*(4,50) = 4.829, *P* = 0.0023). **e**, MGS scores from trainee 2 show significant main effects of time (*F*(4,63) = 9.675, *P* < 0.0001) and treatment (*F*(1,63) = 4.551, *P* = 0.0368), and a significant time × treatment interaction (*F*(4,63) = 3.037, *P* = 0.0236). **f**, Automated MGS scores show a significant main effect of time (*F*(4,50) = 64.57, *P* < 0.0001), no effect of treatment, and no time × treatment interaction. **g**, Distance moved reveals a significant main effect of time (*F*(4,51) = 15.37, *P* = <0.0001), no effect of treatment and a significant time × treatment interaction (*F*(4,51) = 10.33, *P* = <0.0001). **h**, Weight measurements show a significant main effect of time (*F*(5,65) = 3.502, *P* = 0.0073), no effect of treatment and a significant time × treatment interaction (*F*(5,65) = 4.183, *P* = 0.0023). In **c**–**h**, asterisks represent significant Šidák’s post-hoc comparisons; smaller color-coded asterisks report drug versus time effects, and larger black asterisks report between-group effects at a given time point. Error bars represent the s.e.m. *P < 0.05, **P < 0.01, ***P < 0.001, ****P < 0.0001. Icons created in BioRender; Bohacek, J. https://biorender.com/6u7fntj (2026).

In our first test of the GrimACE (experiment 1), we investigated the postoperative analgesic effects of meloxicam and buprenorphine following intracranial surgery. Mice were either unilaterally implanted with two 200-μm optic fibers (one above the locus coeruleus and one within the ventral hippocampus, *n* = 10) or were unilaterally implanted with a single 200-μm optic fiber (above the locus coeruleus, *n* = 5) (Fig. 2a). To comply with the 3R principles, the choice of target sites was guided by ongoing experiments in our lab^43^, so no animals had to be used specifically for the purpose of the experiments reported here. Approximately half of these mice received one dose of meloxicam (M) (5 mg/kg subcutaneously (s.c.)) shortly before surgery (*n* = 8), and the others received three doses of buprenorphine + meloxicam (B + M) (0.1 mg/kg + 5 mg/kg s.c.), once shortly before surgery, once approximately 8 h after surgery and once approximately 24 h after surgery (*n* = 7). Mice were placed in the GrimACE at various time points before and after surgery (see Fig. 2b for the experimental design), and 10-min videos were recorded from front-view and top-view cameras. In addition, mice were manually inspected as part of our postsurgical cage-side assessment routine, and body weight was recorded daily for 3 days following surgery.

#### Cage-side assessment

Routine cage-side assessment could not detect signs of discomfort or pain, as all animals moved normally, their coats were well groomed, they did not show overt postural changes, and wounds were closed and healed normally. On our cage-side assessment scale, which ranges from 0 (normal) to 3 (severely affected) for several measures (see Methods for details and Supplementary Fig. 1 for an example of a cage-side assessment monitoring scale), all animals consistently scored 0 for all measures. This is in line with our extensive experience performing craniotomies in the lab^43–47^ and with the notion that craniotomy leads only to moderate pain levels that can be well managed with M or B + M. However, recent work has clearly shown that signs of pain can be detected even with adequate analgesia regimes in the hours and days following brain surgeries in mice^27,29^. Therefore, we also investigated postsurgical pain using the MGS.

#### MGS scoring

From the front-view video recordings collected in the GrimACE, we manually selected ten frames from each 10-min video for manual grimace scoring by two trainees and one expert. The expert had been scoring and providing training on the MGS for years and had also trained both trainees (see Methods for details). Before the images of the present project were evaluated, trainee 1 had only recently completed the MGS training course (3× ~2-h sessions, with ~2 h of scoring homework). In addition to the training course, trainee 2 had already scored images of C57BL/6J mice and BALB/c mice in two previous projects. In contrast to our cage-side assessment, all raters detected an increase in MGS scores for some animals at the 4-h time point in comparison with the 0-h baseline recordings. Four hours after surgery, both trainees detected significantly higher pain levels in M mice than in B + M mice, while the expert rater did not, although the expert did rate values for M nominally higher than for B + M (Fig. 2c–e). Interestingly, all raters still reported slightly elevated MGS scores, even 48 h after surgery. The automated MGS score, trained on expert-scored frames, was similarly able to detect the surgery-induced peak in pain levels at the 4 h time point (Fig. 2f). It also identified a significant difference between M and B + M at this time point and detected slightly elevated MGS scores in both groups 48 h after surgery. The performance of the different raters and a detailed comparison of the expert rater and the automated MGS score will be discussed in detail later.

#### Pose estimation and body weight

Pose estimation from the top-view camera enabled simple tracking of locomotor activity (raw distance^38^). At the 4-h time point, B + M mice moved significantly more than M mice (Fig. 2g). This is consistent with a large body of literature showing that acute administration of opioids in general, and buprenorphine specifically, induces hyperactivity in mice and rats^48^. Daily body weight measurements revealed a significantly lower body weight for the mice in the B + M group compared with the M group at the 24-h, 48-h and 72-h time points (Fig. 2h). However, this drop in body weight was in the range of 5%, which is far from our termination criterion of 15% body weight loss. The sharp increase in locomotor activity during the acute postoperative period, when rest would be expected, along with the concomitant reduction in body weight, suggests that opioid treatment induces unwanted side effects that probably hinder the recovery process.

#### BehaviorFlow analysis

Top-view pose estimation also enabled more advanced analysis of behavioral motifs and the dynamic transitions between these motifs, using BehaviorFlow^33^. BehaviorFlow was developed with the aim to recognize subtle alterations in the microstructure of mouse behavior, to reveal latent phenotypes that would otherwise remain hidden from human observers. To test whether the two analgesia regimes differentially impacted the animals, we split behavior observations into ten clusters and compared the BehaviorFlow between both groups at every time point (Methods). Analysis indicated that, at the 4-h time point, M and B + M groups showed highly significant differences in their behavior flow (Supplementary Fig. 2a), which is in line with the spike in locomotor activity observed in the B + M group at that time point. For the remaining time points, no differences were detected, indicating that, in agreement with the MGS scores, there were no significant changes in overall behavior between the two treatment groups. We then quantified the time spent in each behavior cluster (Supplementary Fig. 3a). Only cluster 1 showed a significant interaction between treatment and time. In addition, we found significant main effects of time for clusters 5 and 7 in M animals, and significant main effects of time for clusters 1, 5, 6 and 8 in the B + M animals. While the overall BehaviorFlow analysis (Supplementary Fig. 2a) corroborates the observed increase in locomotor activity (Fig. 2g), we see that not every aspect of the animals’ behavior is completely dominated by the increased distance traveled by the B + M mice. This suggests that detailed analyses, such as those presented here, could be more informative for detecting subtle behavioral differences and may help identify stereotyped behaviors associated with pain, such as paw licking or a hunched posture. We provide a detailed evaluation of the clusters in ‘Discussion’.

### Replicated validation of the GrimACE (experiment 2)

**Fig. 3:**
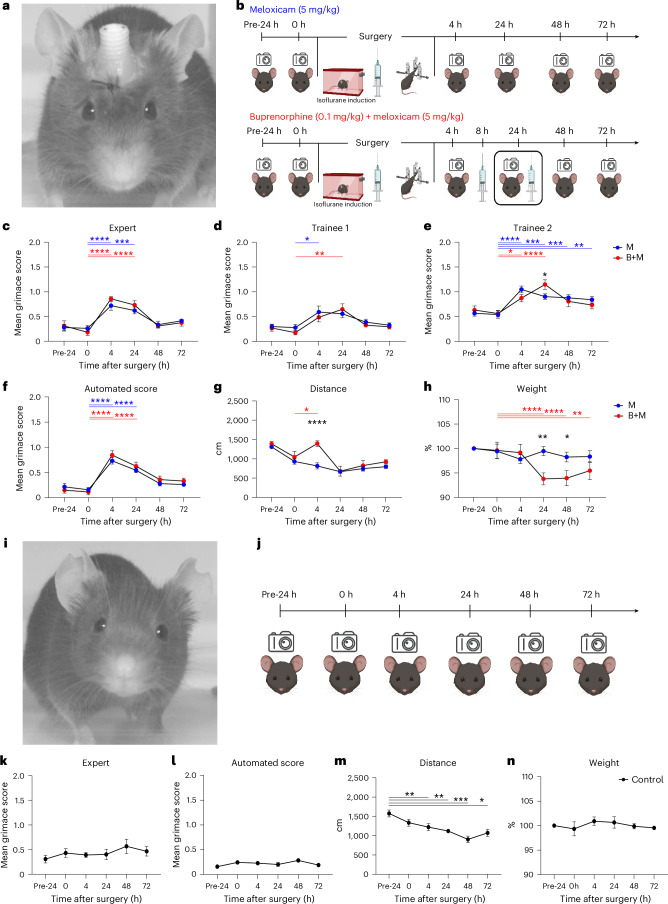
GrimACE assessment after craniotomy with cannula implantation and in a no-surgery control group. **a**, An animal with cannula implants 24 h after surgery. **b**, Experimental timeline showing GrimACE recordings, surgery and injections of analgesia (meloxicam: 10 mice, meloxicam + buprenorphine: 6 mice). **c**, MGS scores from the expert rater show a significant main effect of time (*F*(5,69) = 24.44, *P* < 0.0001), but no effect of treatment and no time × treatment interaction. **d**, MGS scores from trainee 1 also show a significant main effect of time (*F*(5,69) = 7.699, *P* < 0.0001), no effect of treatment, and no time × treatment interaction. **e**, MGS scores from trainee 2 show significant main effects of time (*F*(5,69) = 17.64, *P* < 0.0001) and a significant time × treatment interaction (*F*(5,69) = 2.681, *P* = 0.0284). **f**, Automated MGS scores show a significant main effect of time (*F*(5,69) = 44.80, *P* < 0.0001), no effect of treatment, and no time × treatment interaction. **g**, Distance moved reveals a significant main effect of time (*F*(5,70) = 22.12, *P* < 0.0001), treatment (*F*(1,14) = 7.757, *P* = 0.0146) and a time × treatment interaction (*F*(5,70) = 4.006, *P* = 0.0030). **h**, Weight measurements show a significant main effect of time (*F*(5,70) = 9.813, *P* < 0.0001), no effect of treatment and a significant time × treatment interaction (*F*(1,14) = 1.694, *P* < 0.0001). **i**, A no-surgery control animal in the GrimACE at the 24-h time point. **j**, Experimental timeline showing control GrimACE recordings matched to time points in experiments 1 and 2. **k**,**l**, No significant changes in MGS score were observed by either the expert (**k**) or automated scorer (**l**). **m**, Distance analysis revealed a significant main effect of time (*F*(3.145,22.01) = 12.56, *P* < 0.0001), and individual (*F*(7,35) = 4.456, *P* = 0.0012). **n**, Weight remains stable over time. In **c**–**h** and **k**–**n**, asterisks represent significant Šidák’s post-hoc comparisons; smaller color-coded asterisks report drug versus time effects, and larger black asterisks report between-group effects at a given time point. Error bars represent the s.e.m. *P < 0.05, **P < 0.01, ***P < 0.001, ****P < 0.0001. Icons created in BioRender; Bohacek, J. https://biorender.com/6u7fntj (2026).

We were surprised to observe differences among human raters, and that some noted pain appeared to persist—albeit at a very moderate level—48 h after surgery. This observation contrasts with previous work using the MGS after craniotomy, which found that pain levels return to baseline after 48 h, even without analgesia^29^. We therefore repeated the experiment to validate our findings and again investigated the postoperative analgesic effects of M and B + M following intracranial surgery; however, this time the mice underwent surgery to implant a bilateral hippocampal cannula (Fig. 3a). The reason was that, in keeping with 3R guidelines, we used animals that were scheduled for ongoing experiments in our lab^49^, thus reducing the number of animals needed to test and optimize the GrimACE. We also included an additional monitoring time point 72 h after surgery (see Fig. 3b for the experimental design).

#### MGS scoring

The same three raters again manually scored the extracted frames, and again all raters detected an increase in MGS scores of the animals at the 4-h time point in comparison with the 0-h time point (Fig. 3c–e). Again, MGS scores remained elevated for 24 h. This time, no differences emerged between M and B + M groups, apart from at the 24-h time point according to trainee 2. Trainee 2 also reported that the MGS scores for the M animals were raised at both the 48-h and 72-h time points in comparison with the 0-h time point; however, both the expert and trainee 1 reported that pain levels had returned to baseline at both 48 and 72 h after surgery. The automated MGS score, trained on the expert rater, detected an increase in the MGS score at the 4-h time point in both groups that persisted throughout the 24-h time point, before returning to baseline at the 48-h and 72-h time points (Fig. 3f).

#### Pose estimation and body weight

Similarly to experiment 1, we detected a significant difference in locomotor activity at the 4-h time point between the M and B + M group (Fig. 3g). The weight of the B + M group also dropped significantly at the 24-h and 48-h time points in comparison with the 0-h time point (Fig. 3h). Again, the reduction in body weight was modest and stayed between 5% and 10%, before returning to baseline.

#### BehaviorFlow analysis

BehaviorFlow analysis again showed that the M and B + M groups had significant differences in their behavior flow only at the 4 h time point, indicating that BehaviorFlow differences are in line with the notable spike in locomotor activity (Supplementary Fig. 2b). Detailed analysis of time spent in each behavior cluster again showed a significant interaction between treatment and time only for cluster 1 (Supplementary Fig. 3b). As in experiment 1, this effect was driven by a sharp peak of time spent in cluster 1 at the 4-h time point in the B + M group, but not in the M group. In addition, significant main effects of time emerged for clusters 6, 7, 8 and 10 in M animals, and for clusters 1 and 5 in B + M animals.

### No-surgery control for repeated testing (experiment 3)

The behavioral changes observed in response to buprenorphine injection were dramatic, yet the quantification of different behavior clusters also revealed considerable fluctuations across time points in both treatment groups. To ensure that the MGS and behavioral changes that we observed in experiments 1 and 2 arose from the treatments and not from habituation, we performed a no-surgery control experiment (Fig. 3i). We repeated the exact experimental design of GrimACE exposures across 5 consecutive days as in the previous experiments (Fig. 3j), but without surgery or drug administration (*n* = 8). The results from experiment 3 show that MGS scores remain stable and low across all time points, both when scored by the expert rater (Fig. 3k) and when scored automatically by the GrimACE classifier (Fig. 3j). However, locomotor activity decreased over time (Fig. 3m), similarly to what we observed in M-treated animals in experiments 1 and 2. Body weight remained stable (Fig. 3n). Behavioral clustering revealed some fluctuations of behavior across days, but the clusters that changed significantly over time in the M and B + M treatment groups in experiments 1 and 2 (clusters 1, 6, 7, 8 and 10) did not change significantly over time in this control cohort, which implies that previous observed changes could have been due to their treatment (Supplementary Fig. 3c). While these data suggest that locomotor activity decreases with repeated exposure to GrimACE, the consistent MGS scores and behavioral flow data indicate that habituation to GrimACE is not required for the reliable assessment of pain and well-being.

### Automated versus manual performance

**Fig. 4:**
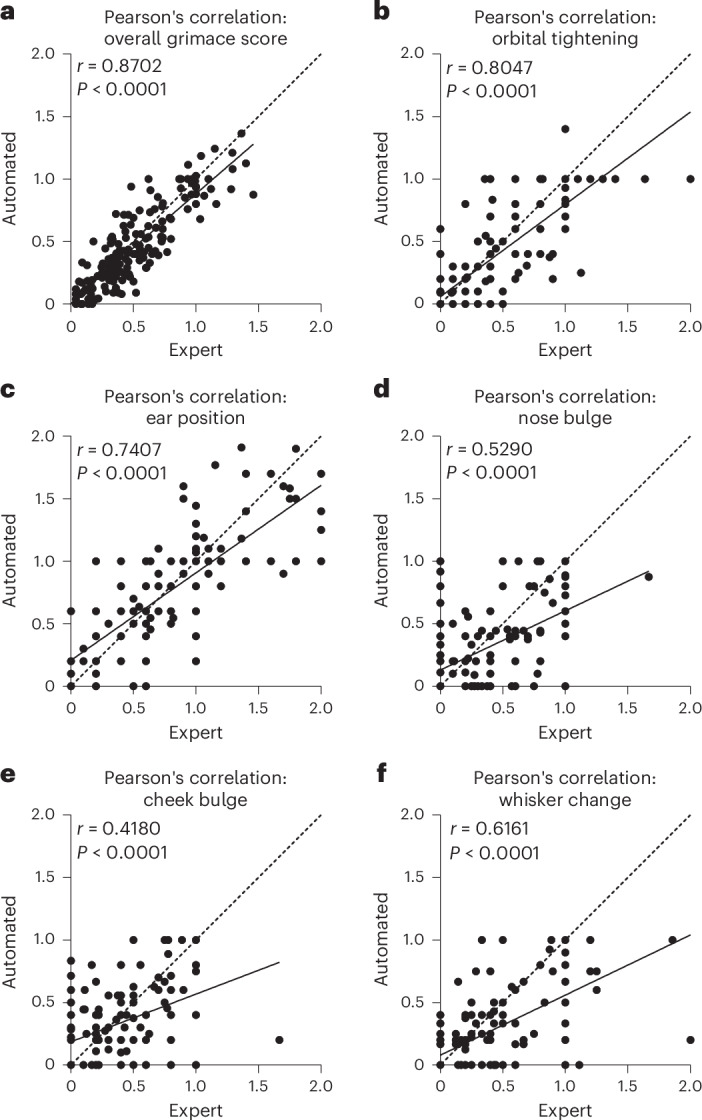
Automated MGS scoring performance. **a**, Mean automated MGS scores for each feature in experiments 1 and 2 show a remarkably high overall correlation with the expert rater (Pearson’s *r* = 0.87). **b**–**f**, Breaking these scores down into their mean components, including orbital tightening (**b**), ear position (**c**), nose bulge (**d**), cheek bulge (**e**) and whisker change (**f**), demonstrates that the neural network is successfully distinguishing individual MGS features, with only cheek correlation having a Pearson’s *r* < 0.5 (**e**). In general, the automated MGS scores are negatively impacted most from a lack of training data with high MGS scores (severe pain levels), particularly for nose, cheek and whisker (**d**–**f**), leading to an MGS score underestimation bias in these areas.

Despite carefully conducted MGS training by the expert (see Methods for details), the trainee raters gave remarkably different MGS scores from the expert and from each other on the same set of images (Supplementary Fig. 4a). For this reason, only the expert rater was used to train the automated MGS scoring network; and indeed, this network showed impressively consistent performance with the expert rater in leave-one-animal-out cross validation (Fig. 4). When assessed on a per-video basis, the overall automated MGS score showed minimal bias across all animals (Fig. 4a). Individual component scores suffered from a lack of high-MGS training examples (that is, few examples in which the feature indicates severe pain and is scored as 2), which led to an overall low-score bias in some component scores (Fig. 4d–f). While we chose to train the network on the data from the expert in these experiments, all raters showed a high degree of self-consistency (Supplementary Fig. 4b–d), which suggests that the MGS scoring network could be trained to replicate the performance of any of these raters. Fine-grained self-consistency of human raters across the individual facial components of the MGS in individual images was also consistent for features such as orbital tightening and ear position but was more highly variable for features such as nose and cheek bulge and whiskers (Supplementary Fig. 5). When comparing the individual feature scores of the expert to the automated rater in the context of experiments 1 and 2, we see that the automated rater performed similarly in the majority of features, with the largest differences being in nose and cheek bulge in experiment 2, which is probably due to the lower intrarater correlation of these features (Supplementary Fig. 6). It is also worth noting that the ear position score remains high following surgery, which could be related to the surgery altering the position of the ears. With more training data, this effect could be circumvented, if it is indeed related to the surgery and not pain. For more detailed frame-level comparisons between the expert and automated scores, see Supplementary Fig. 7.

## Discussion

### Standardization and automation of holistic welfare monitoring

Measuring spontaneous pain and discomfort in laboratory rodents is surprisingly hard^2,19^. Although the use of facial features holds great promise for pain assessment across species, the excitement is hampered by the notion that MGS scores should not be interpreted in isolation^2^, and the fact that MGS scoring is quite difficult and requires substantial investment in training personnel to overcome high inter-rater variability^21,50,51^. Here, we address both problems by combining MGS scoring with in-depth analysis of body posture and behavioral flow, and by standardizing the recording setup to train a machine learning agent on reproducibly high-quality images. We show that the combination of automated MGS scoring with simple movement tracking and advanced behavioral profiling reveals moderate increases in pain scores after craniotomies, when standard cage-side assessment fails to detect overt signs of pain or discomfort, as well as known side-effect profiles of acute opioid administration after surgery. In addition, we observe that the pain scores are slightly higher in experiment 1 (Fig. 2) than in experiment 2 (Fig. 3). While this result could be due to the different implants, it could also be due to less obvious confounders. The surgeries in experiment 2 were performed by a more experienced surgeon, which probably reduced the duration of the surgeries. Experiment 1 was also carried out by a female experimenter, whereas experiment 2 was carried out by two male experimenters, and previous work has shown that this could be the reason for the reduced pain scores observed here^52^.

### Automation of MGS assessment

The automation of MGS scoring can be broken down into two parts: image acquisition automation and image assessment automation. Previous work has focused on the automation of image assessment, and laid the foundations for the present work, by demonstrating that automatic machine learning approaches were feasible, powerful and accurate alternatives to human MGS scoring. Tuttle et al.^22^ used the (then) state of the art InceptionV3 convolutional neural network (CNN) architecture to train a binary classifier to distinguish ‘pain’ from ‘not pain’ and showed that output confidence correlated strongly with total MGS score. Andresen et al.^25^ explored several CNN architectures, with and without pretraining, and achieved a binary classification accuracy of up to 99%. McCoy et al.^24^ took these approaches another step further by embedding CNN-based MGS scoring into a full online PainFace MGS data platform, making these automation approaches more accessible than ever, facilitating the collection of MGS training data and demonstrating impressive performance. The fundamental difference between the GrimACE and these previous works is that it is an end-to-end fully automated system operating in research use, encompassing image acquisition. The standardization of image acquisition might have dramatic impacts on all subsequent steps of MGS assessment. This standardization may contribute to the remarkable self-consistency observed even in trainee MGS raters, despite showing strong differences compared with an expert rater. Consequently, project-specific training of human raters may be beneficial, so that greater consensus can be achieved. Because of these fundamental improvements in training and validation data, we are reluctant to make strong claims about relative accuracy to other automated scoring techniques, although our overall MGS Pearson’s *r* of 0.87 is certainly state of the art. Unlike previous approaches, this accuracy leverages vision transformers, rather than traditional CNN architectures, which permits rapid transfer learning with small training datasets. Generally, however, we emphasize that open-source computer vision tools have reached a level where their application to MGS will primarily be limited by the quantity and quality of training data, and any published network architecture can therefore be expected to improve in performance over time as new training data become available.

The standardized GrimACE images present several advantages, specifically from a machine learning point of view. Most of these advantages stem from the fundamental limitation that, without using standardized images in specialized applications like MGS, it is often infeasible to produce training datasets that adequately sample the covariance of image acquisition setups together with mouse treatments, because training images for different treatments may often be acquired by different labs. This can introduce an inescapable hidden bias that directly impacts the primary goal of many studies: treatment comparisons. Image standardization not only removes this problem, but as a direct consequence, it also dramatically reduces the quantity of training data required for transferable performance. These two issues taken together mean that rapid dissemination of new experimental techniques (that initially are only performed in one lab) can occur in lockstep with standardized automatic pain monitoring, greatly enhancing 3R outcomes. The trade-off, of course, is compatibility with other platforms (for example, PainFace, which requires color images).

### Pain management after craniotomies with head implants

Currently, there are only very few studies that carefully evaluate the appropriate analgesia regime for craniotomies. For a long time, it was thought that craniotomy-induced pain is minimal, and a large number of studies even fail to report their analgesia regime in neuroscience research papers using brain surgeries for virus injections and/or head-implants^28^. Recently, two elegant studies have been published that directly address this issue^27,29^. First, these studies show that craniotomies (with or without placing implants on the skull) induce significant pain levels that are clearly and reproducibly captured by MGS assessment^27,29^. However, these pain levels are moderate (typically around or below an average MGS score of 1), and these pain levels return to baseline, even without analgesia, 2 days after craniotomy^29^. Comparing many different analgesia regimes (including meloxicam (5 mg/kg, s.c.) and buprenorphine (0.1 mg/kg, s.c.)) with a surgery group without analgesia, the investigators show that all analgesia regimes that they used provided similar and effective pain relief compared with non-analgesia. However, similar to our data, pain levels were detectable 4–24 h after surgery. Munk et al.^27^ compared the effectiveness of NSAID-alone (carprofen, 50 mg/kg via drinking water) versus multimodal analgesia (carprofen + buprenorphine depot formulation) after craniotomy with relatively large head implants for electroencephalography recordings. They used MGS in combination with a thorough, multimodal pain assessment to show convincingly that monotherapy with NSAIDs is as effective in managing craniotomy-related pain in mice as NSAID + opioid therapy. Importantly, the slow-release formulation of buprenorphine ensured drug availability throughout the recording period in their study, whereas in our design, buprenorphine was active at the 4-h time point, when pain levels are probably maximal, but not at the subsequent time points as we evaluated pain before the administration of additional analgesia. Thus, together with recent work^27,29^, these results converge on the conclusion that neither NSAID monotherapy nor the combination of meloxicam and the powerful opioid formulations can completely eliminate the remaining, moderate levels of postsurgical pain after craniotomies. In line with previous research, we show that buprenorphine, when administered acutely, causes side effects such as pronounced hyperactivity, which might be detrimental during the recovery phase when rest would be recommended. The key tenet is to strike a balance between sufficient pain management and minimal administration of analgesics, to maximize pain relief while minimizing potential side effects and complications. When taking all the recent evidence into consideration, it appears that, in the acute phase (the first 24 h after surgery) NSAIDs at moderate doses (meloxicam 5 mg/kg or carprofen 10 mg/kg) provide pain relief to some extent, and the addition of opioids provides either no or only very little additional analgesic effect. However, these findings also suggest that there might be opportunities to improve analgesia after craniotomy and further reduce pain levels. Given that potent depot formulations of buprenorphine could not achieve additional pain relief, it is also possible that pain management cannot be improved beyond this point. Although this work focuses on pain management following craniotomies with head implants, it is also important to note that the GrimACE could also be used to investigate and assess pain and pain management in other scenarios and models of acute and chronic pain, such as in nerve damage or injuries^53,54^. In such models, the ability to assess behavioral features beyond grimacing, for example the emergence of repetitive behaviors such as paw licking or hunched body posture, will probably prove beneficial, as discussed in the next section.

### BehaviorFlow

A strength of the GrimACE is that it enables a detailed profiling of behavioral changes based on whole-body pose estimation, extending pain and welfare assessment far beyond the assessment of facial features. A more detailed examination of the clusters identified by the BehaviorFlow analysis reveals that they are composed, at least in part, of identifiable behaviors, including grooming (cluster 2), clockwise or counterclockwise rotation (clusters 3 and 10), rearing and sniffing (clusters 5, 6, and 9), immobility (clusters 1 and 8), head movement (cluster 7) or a combination of these behaviors (cluster 4). By looking at the clusters that change over time in experiment 3 (Supplementary Fig. 3), we can identify clusters that are involved in baseline habituation to the GrimACE. These include cluster 5, which contains large amounts of exploratory sniffing and interacting or rearing against the arena. Time spent in cluster 5 seems to be high upon first exposure to the box, then much lower when the animals are in pain, or during subsequent exposures to the arena. This finding suggests that exploratory behaviors decrease as the animal becomes familiar with the environment^55,56^. Conversely, cluster 8 contains a type of immobility/inactivity that seems to increase with exposure to the GrimACE but decreases in the B + M group at a time when mice exhibit hyperactivity. Meanwhile, clusters 1, 6, 7 and 10 seem to be related to the treatment groups. Cluster 1 is highly enriched at the 4-h time point in B + M mice, which is strange as it seems to contain yet another type of immobility/inactivity and the B + M mice are hyperactive at this time point. However, careful visual inspection of this cluster in both groups suggests that the B + M mice move more in short bursts, as if they wanted to settle but feel compelled to move frequently, whereas M mice spend more time in a different immobility cluster (cluster 8) at this time point. Cluster 7 seems to be more variable following M treatment and could indicate more head movement or head-specific grooming, specifically at the 4-h time point. Cluster 6 changes over time following both M and B + M treatment, which is no surprise as it contains a relatively high amount of rearing behavior that is probably more difficult for the mice to perform following surgery.

### Limitations

Due to the experimental requirements of our laboratory, our approach focuses on brain surgeries and on C57BL/6 mice, which represent the most commonly performed surgical procedures and, by far, the most frequently used mouse strain in neuroscience laboratories worldwide^1,57^. The current experiments were conducted only in males, because these data were collected from ongoing experiments in the lab that happened to be conducted in males. However, previous papers that used MGS in both sexes found very similar pain scores and also a very similar response to NSAID and buprenorphine injections^27,29^. In addition, we tested our setup only on animals that received effective analgesia regimes; thus, pain levels were low across animals, and we do not have sufficient training data on more severe pain levels. Therefore, our setup and analysis pipeline can be readily implemented by other laboratories, but the algorithm would have to be trained and validated further on both sexes and on experimental conditions with more severe pain levels.

Our automated MGS scoring system has been designed for maximum data exploitation and flexibility; therefore, it contains no hard-coded face-region biases. This means that the automated MGS scores can, even without sufficient training data, also take into account relevant facial features that are not codified into the MGS, such as the presence of an implant, stitches, hair loss or wet fur. We see that, through sufficient context-relevant training data, the network successfully and accurately detected the decrease in postsurgery MGS despite the constant presence of a head implant; however, for accurate results in other contexts, additional training would be required. In the case of more visually variable conditions, such as hair loss or overgrooming, large training datasets would be required to provide reliable grimace scores, and it may be more effective to implement specific analyses to detect and measure hair loss. Currently, we have validated the GrimACE only with C57BL/6 mice; however, with adequate training data, it would be possible to use it with other visually distinct strains of mice (white, nude and so on). If the physical setup does not provide high-quality raw data when working with other mouse strains (for example, white mice on a white background), the modular design of the system allows higher-contrast materials to be used easily. However, based on our experience with white mice in other testing setups, this may not be necessary.

It is also hoped that, in the future, a standardized welfare monitoring system such as this could be incorporated into the animals’ home cages, allowing assessment in a less intrusive manner and within a more ethologically relevant environment. However, given the current need for advances in cage-side assessment and the difficulty of implementing this type of holistic welfare assessment in the home cage, a system such as this one can provide an immediate improvement in animal welfare.

## Methods

### Animals

To comply with the 3R principles, mice for these experiments were used on the basis of ongoing experiments in our lab^43,49^. The cohorts assessed in these experiments happened to be males; thus, we do not have data for female animals. DBHiCre mice on a C57BL/6 background ^45^ (2–3 months of age) (experiment 1 + 2) and C57BL/6 mice (2–3 months of age) (experiment 3) were bred in house. Mice were maintained in a temperature-controlled (22 °C) and humidity-controlled (52% relative humidity) OHB facility under a 12-h reversed light–dark cycle (lights on at 8:15), with food (M/R Haltung Extrudat, Provimi Kliba SA, cat. no. 3436) and water provided ad libitum. Mice were housed in individually ventilated cages (SealSafe PLUS GM500, Tecniplast) in groups of three to five per cage and used for experiments when 2–3 months old. Cages contained wood chip bedding (LIGNOCEL SELECT, J. Rettenmaier & Söhne) nesting material (tissue paper) and a red acrylic shelter. For each experiment, mice of the same age were used in all experimental groups to rule out confounding effects of age. All tests were conducted during the animals’ active (dark) phase from 9:00 to 18:00. All procedures were carried out in accordance with Swiss cantonal regulations for animal experimentation and were approved under licenses ZH067/2022 and ZH001/2021.

### Stereotaxic surgeries

For experiment 1 (Fig. 2) 15 male DBHiCre mice at the age of 2–3 months were subjected to stereotactic surgery. The mice were anesthetized with 4% isoflurane (Attane, Piramal Pharma Limited) in a transparent induction chamber and then placed in a stereotaxic frame with continuous anesthesia via nosecone of 2% isoflurane. For analgesia, animals in the meloxicam group received a single subcutaneous dose of meloxicam (Metacam, Boehringer-Ingelheim) in sterile saline (5 mg/kg) immediately after isoflurane induction, whereas animals in the meloxicam + buprenorphine group received three subcutaneous injections of meloxicam + buprenorphine (Temgesic, Temgesic solution, 0.3 mg/ml, Reckitt Benckiser) in sterile saline (5 mg/kg and 0.1 mg/kg, respectively), one immediately after isoflurane induction and the other two approximately 8 h and 24 h after surgery. Both groups received local analgesics lidocaine (2 mg/kg) (Lidocaine 1% Streuli, Streuli Pharma) and bupivacaine (2 mg/kg) (Bupivacain Sintetica, Sintetica) topically applied at the site of the wound before incision. In addition, vitamin A cream (Bausch + Lomb) was applied to the eyes to stop them from drying out during the surgical procedure. After the skull was exposed, bregma (defined as the intersection of the coronal and sagittal suture) was located and the skull placement was corrected for tilt and scaling. Holes were then drilled either above the right ventral hippocampus (rvHC) −3.3 mm anterior–posterior (AP), 3.2 mm medial–lateral (ML) from bregma (*n* = 5); or above the rvHC and the locus coeruleus −5.4 AP, 0.9 ML from bregma (*n* = 10). Mice were then injected with either 0.25 µl ssAAV-9/2-hSyn1-mCherry-WPRE-hGHp(A) 7.5 ×10^12^ vg/ml (*n* = 5 rvHC only −3.3 mm AP, 3.2 mm ML, −3.8 mm dorsal–ventral (DV)), or 0.25 µl of ssAAV-5/2-hEF1α/hTLV1-dlox-ChrimsonR_tdTomato(rev)-dlox-WPRE-bGHp(A) 4.7 × 10^12^ vg/ml in the locus coeruleus (−5.4 AP, 0.9 ML, −3.8 mm DV) and ssAAV-9/2-hSyn1-GRAB_NE2m-WPRE-hGHp(A) 2.11 × 10^12^ vg/ml or ssAAV-9/2-hSyn1-EGFP-WPRE-hGHp(A) 2.9 × 10^13^ vg/ml in the rvHC (−3.3 mm AP, 3.2 mm ML, −3.8 mm DV) using a pneumatic injector (IM-11-2, Narishige) and calibrated microcapillaries (cat. no. P0549, Sigma-Aldrich). An optical fiber/optical fibers (200 µm, numerical aperture 0.37; Neurophotometrics) were then implanted 200 μm above the injection coordinates. Optical fibers were affixed to the skull using a bonding agent (Etch glue, Heraeus Kulzer) and an ultraviolet-curable dental composite (Permaplast LH Flow; M + W Dental). Stitches (Supramid Polyamide pseudo monofilament, non-absorbable, DS19; B. Braun) were applied as needed. The health of all animals was monitored over the course of 3 consecutive days after surgery using manual cage-side assessment (see below).

For experiment 2 (Fig. 3), 15 male DBHiCre mice at the age of 2–3 months were subjected to stereotactic surgery. The mice were anesthetized with 4% isoflurane and then placed in a stereotaxic frame with continuous anesthesia of 2% isoflurane. For analgesia, animals in the meloxicam group received a single subcutaneous dose of meloxicam (5 mg/kg) immediately after isoflurane induction, whereas animals in the meloxicam + buprenorphine group received three subcutaneous injections of meloxicam + buprenorphine (5 mg/kg and 0.1 mg/kg respectively), one immediately after isoflurane induction, and the other two approximately 8 h and 24 h after surgery. Both groups received local analgesics lidocaine (2 mg/kg) and bupivacaine (2 mg/kg) at the site of the wound before incision. In addition, vitamin A cream (Bausch + Lomb) was applied to the eyes to stop them from drying out during the surgical procedure. After the skull was exposed, bregma (defined as the intersection of the coronal and sagittal suture) was located and the skull placement was corrected for tilt and scaling. Bilateral holes were drilled above the hippocampus at −1.8 mm AP and ±1.5 mm ML from bregma, followed by the implantation of a bilateral guide cannula (62036, RWD Life Science) into the dorsal hippocampus (coordinates from bregma: −1.8 mm AP, ±1.5 mm ML, −1.5 mm DV). The health of all animals was monitored over the course of 3 consecutive days after surgery.

### Manual grimace scale scoring

To establish the ground truth and generate training data that could later be used to automate the grimace scoring process, we first asked three trained experimenters to score 5–15 images from each video and record their grimace scores. Orbital tightening, nose bulge, cheek bulge, ear position and whisker change were all scored either 0 (not present), 1 (moderately present) or 2 (obviously present). The raters were identified as expert, trainee 1 and trainee 2. The expert ran a grimace scale training course that both trainees attended. In this course, participants spent three (approximately) 2-h sessions scoring images together with the expert and other course participants, learning how to identify the different scores for each feature and reaching a consensus on how each feature should be scored. Participants in this course also learned to identify images showing behaviors such as sniffing, grooming and food intake, so that these could be excluded from scoring, as they can affect the accuracy of the grimace scale. Between each course session, participants completed approximately 1 h of additional scoring as homework. While both trainees attended this course, trainee 2 had slightly more experience than trainee 1 (that is, she had already scored images of C57BL/6J mice and BALB/c mice in two previous projects). For the scoring process, a spreadsheet and folders containing the images were provided to the raters. All raters were blinded to the group assignment, so they did not know which animal received which analgesia protocol nor which time point of the experiment they were scoring. However, the presence of head implants makes it apparent whether an image was taken before or after surgery. In some images, wet fur on the mice’s faces further complicated scoring.

### Manual cage-side assessment scale

The postoperative score sheet assessments range from 0 (normal) to 3 (severely affected) for several measures (see Supplementary Fig 1 for an example of the cage-side assessment monitoring scale). All animals consistently scored 0 on all measures. Experiment 1 was performed by a female experimenter, while experiment 2 was performed by two male experimenters. Experiment 3 was performed by both male and female experimenters.

### The GrimACE

The GrimACE (Fig. 1) is a complete hardware and software solution for standardized MGS image acquisition, scoring and key point-based pose estimation. It consists of an aluminum extrusion frame with custom 3D-printed (PLA) components to hold lights (custom IR lighting from RS Textiles), cameras (2× Basler aca-1300-60gm, 1× Computar S-Mount H0320kp lens with S-mount adapter and 1× Computar 8 mm C-Mount M0814MP2 lens) and an acrylic arena (internal dimensions 11 × 7 × 6.5 cm, with an IR-permeable front/lid and matte white walls). To improve standardization and image quality, matte white acrylic shields have been attached to the frame. The cameras are connected to a laptop (minimum specifications: Intel i7 cpu, NVIDIA 4070 RTX) and powered using a Netgear GS305P-200PES POE switch using 3 GigE cables. The IR light has its own 12-V power supply. Once the GrimACE has been assembled and all powered components are switched on, the user should open the GrimACE app and ensure that both cameras are connected properly. Next, the user should set the working directories and ensure they know which prefixes they will add to the file names for file identification at a later stage. The user may also edit settings such as the duration of the recording and the frame scoring interval. The user should then slide the arena out of the setup, remove the lid of the arena and place a mouse into it. The lid should be carefully closed so as to not trap the tail of the mouse. Once the mouse is safely placed in the arena, it should be gently positioned as shown in Fig. 1b. The user may now click start recording. As some of the components of the GrimACE are from external parties and may be discontinued, it is advised that anyone interested in using the system should contact the 3R hub at ETH Zurich (https://ethz.ch/en/research/ethics-and-animal-welfare/animal-experimentation/3r-principles/3r-hub.html), who can help with the acquisition of parts, assembly, calibration and usage of the GrimACE.

### Pose estimation and BehaviorFlow analysis

Pose estimation was performed using DeepLabCut^58^ while the GrimACE was being developed, and this was used for the analysis above. However, GrimACE now uses YOLO^39^ to provide real-time pose estimation. Pose estimation data from the GrimACE were then processed as described in refs. ^33,38^. In short, 41 features were computed on the basis of DeepLabCut tracking of both mice and grimace box, including acceleration of points, angle between two point pairs, distance between two points and to nearest border, and area spanned by multiple points. These features were then normalized and expanded over ±15 frame sequences. To perform *k*-means clustering, we selected 18–20 recordings from each experiment at random, resulting in 56 recordings in total. The computed feature set of each frame for these recordings was then segmented into ten clusters. A neural network was trained on these clustering results to transfer the clustering to the remainder of the recordings. Before running the behavior flow analysis, cluster labels were smoothed within a five-frame window, followed by computing transition numbers between clusters for each animal. These transition matrices were used for the BehaviorFlow analysis, which assesses overall differences across all transitions. A separate BehaviorFlow analysis was performed at each time point comparing cluster transitions between the M and the B + M group.

### Statistical analysis

Statistical analysis was performed using Graphpad Prism 10 and R studio. In Figs. 2c–h and 3c–h, a mixed-effects model (restricted maximum likelihood) was used to determine fixed effects of ‘Time’, ‘Treatment’ and ‘Time × Treatment’. In Fig. 3k–n, a repeated-measure one-way analysis of variance was used to determine the fixed effects of time and individual. When these effects were determined to be significant (*P* < 0.05), Šidák’s/Tukey’s multiple comparison test was used to further investigate the effects. Asterisks represent significant Šidák’s/Tukey’s post-hoc comparisons; smaller color-coded asterisks report drug versus time effects, and larger black asterisks report between-group effects at a given time point. *P < 0.05, **P < 0.01 ***P < 0.001, ****P < 0.0001. In Supplementary Fig 2, asterisks represent one-tailed *z*-tests. In Supplementary Fig 3, asterisks represent significant main effects of time in analyses of variance. *P < 0.05, **P < 0.01, ***P < 0.001, ****P < 0.0001. Error bars represent the standard error of the mean (s.e.m.).

### Automated MGS scoring workflow

The MGS scoring workflow operates on contiguous segments of front camera video with a user-specified interval (for example, 10 s). Each segment of video is passed to a model that consists of three neural networks. First, a frame quality detection network selects a single frame (image) from the video segment where the mouse face is clearly visible. Next, a mouse face bounding box network detects the mouse face and outputs a bounding box indicating its location in the image. Then, the image is cropped around the bounding box and passed to the MGS network. The MGS network is a classifier that scores each of the orbital tightening, nose bulge, cheek bulge, ear position and whisker change as an MGS value of 0, 1, 2 or ‘not rateable’.

### Frame-quality detection network

The frame-quality detection network was trained using 3,200 images from experiments 1 and 2, which were manually scored for image quality, based on the mouse face being visible, facing toward the camera and not being blurred by motion. Each image was assigned an overall frame-quality score between 0 and 6. The model used PyTorch Image Models^59^ mobilenetv3_large_100 with default pretrained weights feeding into a single output with sigmoid activation. The model outputs a continuous, normalized frame quality score between 0 and 1, which is then multiplied by 6 to return it to the original frame quality scale between 0 and 6. The model was trained using Adam optimization with learning rate 0.0001 and no weight decay, and the following training augmentations: horizontal flip (*P* = 0.5); uniform random shift in brightness (±15%) and contrast (±5%).

### Mouse face bounding box network

A total of 1,076 images from the GrimACE front camera, acquired during experiments 1 and 2 as well as other unrelated experiments, were manually labeled with bounding boxes around the mouse’s face. These data were split into a tenfold cross-validation dataset without splitting images from individual animals across folds. These training and validation sets were then used to train ten Ultralytics YOLO v8s models with default parameter and weight settings^39,40^. In subsequent steps, networks were chosen that were not trained using the validation animal (see below).

### MGS network

The MGS network training data consists of 1,245 expert-scored images from the 31 mice used in experiments 1 and 2, with a mean of 40.16 training images per mouse (standard deviation 12.24). Because the available training data were from experiments 1 and 2, MGS network training and validation differed slightly between these experiments and experiment 3. The MGS network training and validation for experiments 1 and 2 was conducted with leave-one-animal-out cross-validation across the 31 animals from those experiments. That is, the entire MGS network was retrained from scratch and validated for each of these 31 animals independently, using only the data from the other 30 animals. The MGS network for experiment 3 was trained on all the available training data for a fixed number of epochs, based on the observed performance of the networks for experiments 1 and 2.

The training set images for each trained MGS network underwent content augmentation by a factor of 300%: the frames immediately preceding and following the labeled training frame were added to the training set with the same MGS target values (under the assumption that the MGS score varies little within 1/30th of a second). The validation set images were not augmented in this way. Each training and validation image was then run through the mouse face bounding box network, and the image, bounding box and target MGS score were input to the MGS network training.

The MGS network used the Pytorch Torchvision vit_b_16 vision transformer network as a base^41,42^. Vision transformers show exceptional ability to efficiently leverage pretrained weights in image classification tasks^41^. We selected the versatile IMAGENET1K_V1 pretrained weights within Pytorch Torchvision for good network flexibility in subsequent fine tuning. On top of the vision transformer, two fully connected hidden layers were added with ReLU activation, having sizes of 2,048 and 1,024 neurons and input dropout rates of 0.4 and 0.2, respectively. The network output had five linear activation heads (corresponding to orbital tightening, nose bulge, cheek bulge, ear position and whisker change) each of size 4 (corresponding to MGS of 0, 1, 2 and ‘not rateable’).

During training, error was scored with a composite cross-entropy loss: the sum of the cross-entropy loss for each of the five output heads. The optimization was performed using the Pytorch stochastic gradient descent module (SGD) with initial learning rates of 0.0001 for the base vision transformer, and 0.001 for the hidden and output layers. The learning rates decayed by a factor of 0.1 every five epochs. A weight decay of 0 was applied to all weights, and a momentum of 0.95 was used in the SGD. Training ran for 20 epochs using a batch size of 16. Network performance plateaued after ten epochs, so the network state after ten epochs was used for all results.

### MGS network: image preprocessing and training-time augmentation

Because the base vision transformer was trained on Imagenet 1K color images, all our 1,280 × 1,024 grayscale input images were first broadcast to 1,280 × 1,024 × 3 by replicating the grayscale pixel values into each of the three ‘color’ channels. The training dataset images then underwent stochastic preprocessing and training-time augmentation, and the validation set images only underwent deterministic basic pre-processing.

### MGS network: validation data preprocessing

The validation data were not subjected to any random processes or augmentation. First, the bounding box was expanded by 5% about the box center and then squared by increasing the smallest box dimension about the center. The image was then cropped to the bounding box, with zero padding where necessary (that is, if the bounding box crosses the edge of the image). The cropped image pixel values were normalized using fixed mean and standard deviation calculated across the entire dataset of padded, squared, cropped images. Finally, the normalized image was resized to match the input size of the vision transformer (224 × 224 × 3) using bilinear interpolation.

### MGS network: training data preprocessing and augmentation

For each presentation of a training image during training, the following steps were performed. The mouse face bounding box was first randomly adjusted to account for fluctuations from the mouse face detection network. For each bounding box, either each boundary of the box was randomly expanded/contracted by up to 10% (probability 0.5), or each boundary of the bounding box was expanded by 5% in each dimension around its center (probability 0.5). The bounding box was then made square by growing the smallest dimension about the center to match the largest dimension. The image was then cropped to the bounding box, and zero-padded if the bounding box crossed the image edge, to avoid introducing location bias. After cropping, the image was flipped horizontally with probability 0.5. The brightness and contrast were then randomly adjusted within the range ±15% (brightness) and ±5% (contrast). The image was then either: with probability 0.25 rotated by a random angle in the range −10° to +10° and interpolated back to the pixel grid linearly; with probability 0.25 rotated by a random angle in the range −10° to +10° and interpolated back to the pixel grid using the nearest pixel value; or with probability 0.5 passed through with no rotation or interpolation. The cropped image pixel values were normalized using fixed mean and standard deviation calculated across the entire dataset (same values as for validation images). Finally, the image was rescaled to match the input size of the vision transformer (224 × 224 × 3) by interpolating either bilinearly or with nearest pixel value, each with probability 0.5.

### Frame selection

In experiment 1, frames were selected manually for scoring. In experiment 2, SLEAP^60^ was trained to track key points on the nose and ears of the mouse from the front camera. Frames where any of the nose and ear key points had confidence <0.5 were excluded. Next, a binary classifier was trained on 2,610 frames manually scored as either suitable for MGS or not suitable for MGS. The model used PyTorch Image Models^59^ densenet121 with default pretrained weights and a single sigmoid output node, Adam optimization, learning rate 0.0005, weight decay of 0.001 and no data augmentation. Frames with confidence <0.5 according to this method were excluded. Frames with the highest confidence were then selected according to the following criteria: each selected frame was at least 80 frames apart from the others, and no more than 5 frames were chosen per video. In experiment 3, frames were selected using the continuous frame quality detection network. From each 10-s segment of video, the frame predicted to have the highest score was selected, with a minimum quality score threshold of 4.25. In a 10-min testing period, one can expect approximately 25 usable frames. In the experiments for this Article, only 3 out of 225 recordings contained no usable frames; notably, these were all at later time points, which indicates that habituation to the arena could lead to fewer frames being collected owing to reduced exploration of the arena.

### Reporting summary

Further information on research design is available in the Nature Portfolio Reporting Summary linked to this article.

## Online content

Any methods, additional references, Nature Portfolio reporting summaries, source data, extended data, supplementary information, acknowledgements, peer review information; details of author contributions and competing interests; and statements of data and code availability are available at 10.1038/s41684-026-01695-9.

## Supplementary information

Supplementary Information Supplementary Figs. 1–7.

Reporting Summary

## Data Availability

All data, software and hardware designs are available via GitHub at https://github.com/ETHZ-INS/GrimACE_manuscript and via Zenodo at 10.5281/zenodo.15119195 (ref. ^61^) or upon request. Please note that the GrimACE software is compatible only with the GrimACE hardware.
