# Supplementary material for: GrimACE: automated, multimodal cage-side assessment of pain and well-being in mice
Source: Lab Anim (NY). 2026 Mar 5;55(4):137–46. doi: 10.1038/s41684-026-01695-9 (PMC13043301; doi:10.1038/s41684-026-01695-9)
Supplement: Supplementary file 1 — Supplementary Figs. 1–7. [file 41684_2026_1695_MOESM1_ESM.pdf]

lab animal

https://doi.org/10.1038/s41684-026-01695-9

GrimACE: automated, multimodal cage-side assessment of pain and well-being in mice

In the format provided by the authors and unedited

ANNEX 1 (back)

POST-OPERATIVE MONITORING

Mouse Monitoring Scale

Scale	0	1*	**2	***3
Hair Coat	Normal	Rough, apparent lack of grooming	Thin and/or wounded	Wound with discharge
Activity	Normal	Isolated, abnormal posture	Huddled, inactive or overactive	Moves only when touched
Movement	Normal	Slightly uncoordinated or abnormal	Uncoordinated, reluctant to move	Staggering, paralysis, limb dragging
Mouse body condition scoring	Normal	-	Underconditioned, dorsal pelvic bones readily palpable	Emaciated, vertebrae distinctly segmented
Body Weight	Normal	5% loss in 24 h	5-10% loss in 24 h[$] OR 15% overall loss	>10% loss in 24 h OR >15% overall loss
Surgery site/wound	Normal, healing	Swollen/red	discharge or gap formation	Severe bleeding, wound reopening, reddening
Dehydration[#]	Normal	-	Skin tents	Sunken eyes

[#]Dehydration will only be measured if one of the other scores is 1 or higher.

[$] if the animal loses 5-10% of body weight in 24hrs, it will receive a s.c. injection of warm sterile saline (10ul/g)

* with >2 scores of 1 mice will receive an injection of analgesic (Meloxicam, 5mg/kg s.c, 10ul/g). If >2 scores of 1 persist for more than 3 days, mice will be euthanized.

**Score 2 will be tolerated for maximum one day, mice will receive 5mg/kg Meloxicam s.c. every 12 hours, and an additional injection of buprenorphine (0.1mg/kg s.c., 10ul/g) if pain relief is necessary within less than 12 hours of the last meloxicam injection.

***Animals showing score 3 signs will be euthanized immediately.

Appendix 2 (back)

POST-OPERATIVE MONITORING

Mouse Monitoring Scale

Scale	0	1	2	3
**Hair Coat**	Normal (well-groomed coat, clean appearance)	Rough, apparent lack of grooming	Thin and/or wounded	Wound with discharge
**Activity**	Normal (runs, climbs, grooms, interacts with cage mates)	Isolated, abnormal posture	Huddled, inactive or overactive	Moves only when touched
Eyes: Orbital Tightening^	Orbital Tightening Score = 0	Orbital Tightening Score = 1	Orbital Tightening Score = 1	Orbital Tightening Score = 2
**Movement**	Normal	Slightly uncoordinated or abnormal	Uncoordinated, reluctant to move	Staggering, paralysis, limb dragging
**Mouse body condition scoring**	Normal	-	Underconditioned, dorsal pelvic bones readily palpable	Emaciated, vertebrae distinctly segmented
**Surgery site/wound**	Normal, healing	Swollen/red	discharge or gap formation	Bleeding, wound reopening.

^ = Orbital tightening will be assessed as described in Langford et al, 2010, Nature Methods (doi:10.1038/nMeth.1455) on a visual scale from 0 to 2. If no orbital tightening is present the score = 0. If moderate orbital tightening is present the score = 1. If severe orbital tightening is observed the score = 2.

Supplementary Figure 1, Post Operative Monitoring Scale from license ZH001/21 and ZH067/22.

Experiment 1: Fiber optic implant

a)

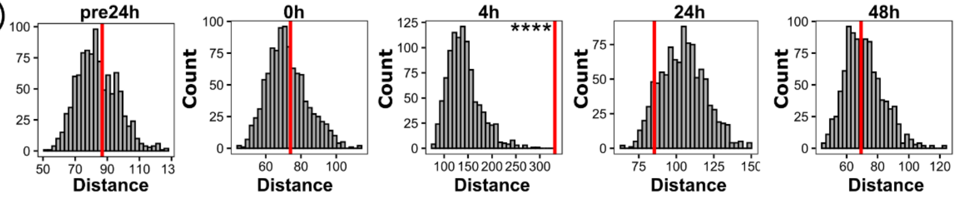

Experiment 2: Cannula implant

b)

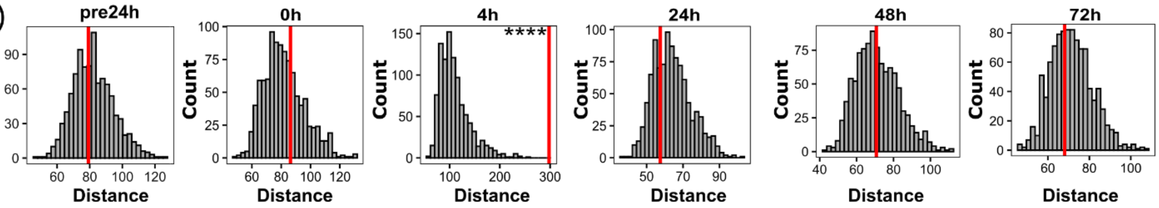

Supplementary Figure 2: Behavior flow analysis of experiments 1 and 2. (a) Behavior flow analysis following craniotomy and fiber optic implantation showed no significant difference between M and B+M animals at the pre24 0h, 24h or 48h, but a significant difference between treatment groups at the 4h timepoint ($p<0.0001$) implant. (b) Behavior flow analysis following craniotomy and bilateral cannula implantation showed no significant difference between meloxicam and buprenorphine + meloxicam animals at the pre24, 0h, 24h, 48h or 72h time points, but a significant difference between treatment groups at the 4h time point ($p<0.0001$). Asterisks represent one-tailed *z*-tests, ****=$p<0.0001$.

Experiment 1: Fiber optic implant

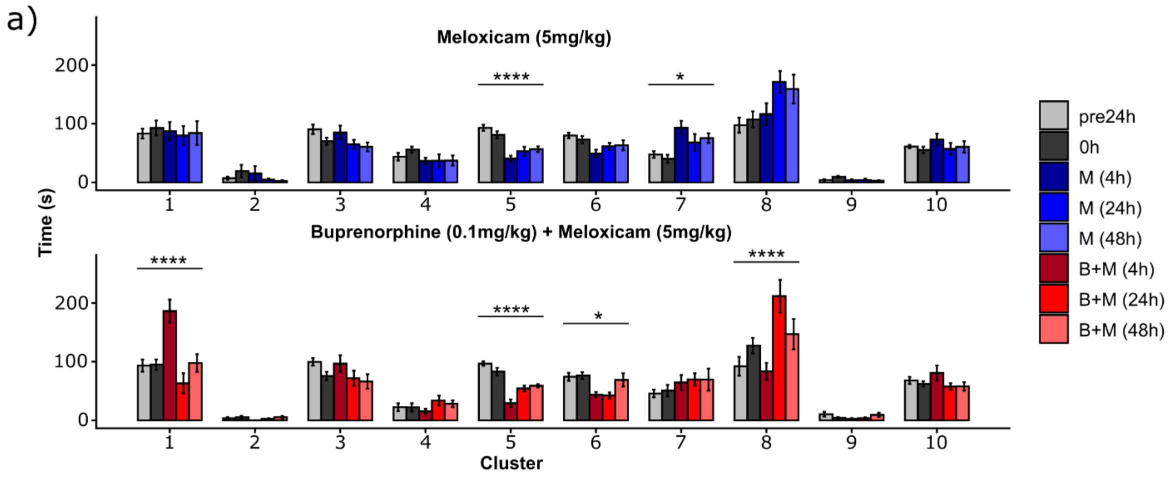

Experiment 2: Cannula implant

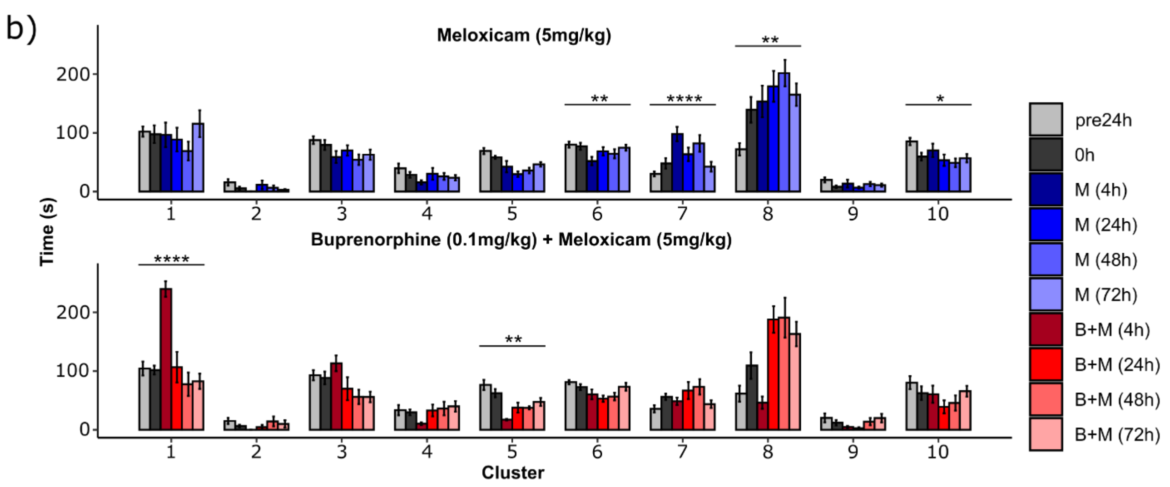

Experiment 3: No surgery control

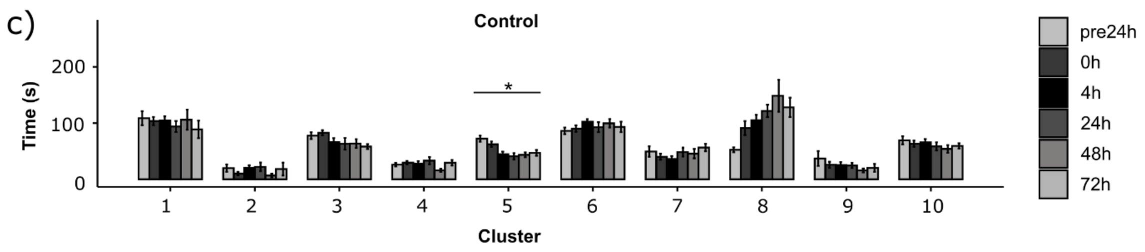

Supplementary Figure 3: Time in cluster analysis for experiments 1,2 and 3. (a) Analysis of time spent in behavioral clusters following craniotomy and fiber optic implantation revealed a significant interaction between treatment and time in cluster 1 (2-way repeated measures ANOVA with multiple testing correction, $F_{,(4,48)}=5.236$, adj $p=0.03$),a significant main effect of time for clusters 5 and 7 for M animals ($F_{,(4,24)}=15.36$, adj $p<0.0001$; $F_{,(4,24)}=5.219$, adj $p=0.04$), and a significant main effect of time for clusters 1,5,6 and 8 in B+M animals ($F_{,(4,24)}=14.762$, adj $p<0.0001$; $F_{,(4,24)}=31.363$, adj $p<0.0001$; $F_{,(4,24)}=5.659$, adj $p=0.002$; $F_{,(4,24)}=13.196$, adj $p<0.0001$). (b) Analysis of time spent in behavioral cluster following craniotomy and bilateral cannula implantation reports a significant interaction between treatment and time only for cluster 1 (2-way repeated measures ANOVA with

multiple testing correction, $F(5,70)=6.86$, adj $p=0.000876$), a significant main effect of time in clusters 6, 7, 8 and 10 for M animals ($F,(5,45)=4.75$, adj $p=0.01$; $F,(5,45)=9.993$, adj $p<0.0001$, $F,(5,45)=5.435$, adj $p=0.0054$, $F,(5,45)=9.993$, adj $p<0.0001$), and a significant main effect of time in clusters 1 and 5 in B+M animals ($F,(5,25)=14.357$, adj $p<0.0001$; $F,(5,25)=12.41$, adj $p=0.00429$). (c) Time-in-cluster analysis in the no-surgery no-analgesia control animals shows significant changes for cluster 5 ($F(2.9,17.41=13.034$, $p=0.000115$). Asterisks represent significant main effects of time in ANOVAs. *=p<0.05, **=p<0.01, ***=p<0.001, ****=p<0.0001.

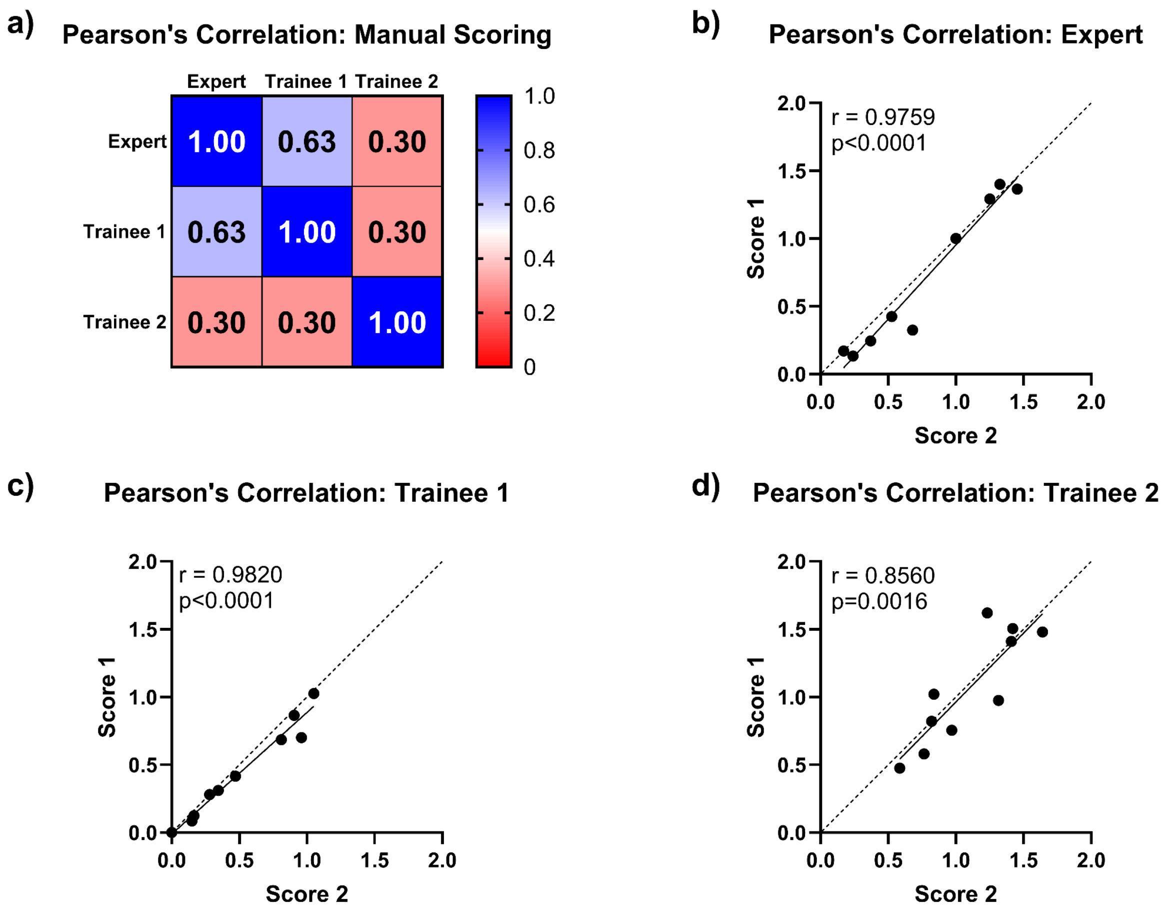

Supplementary Figure 4: Inter-rater and intra-rater correlations for manual MGS scoring. (a) MGS scores from the three human raters show low inter-rater correlations. (b-d) Each rater scored the same 10 images twice (blinded), and the results showed highly significant intra-rater correlations, showing that each rater scored the same images with high consistency.

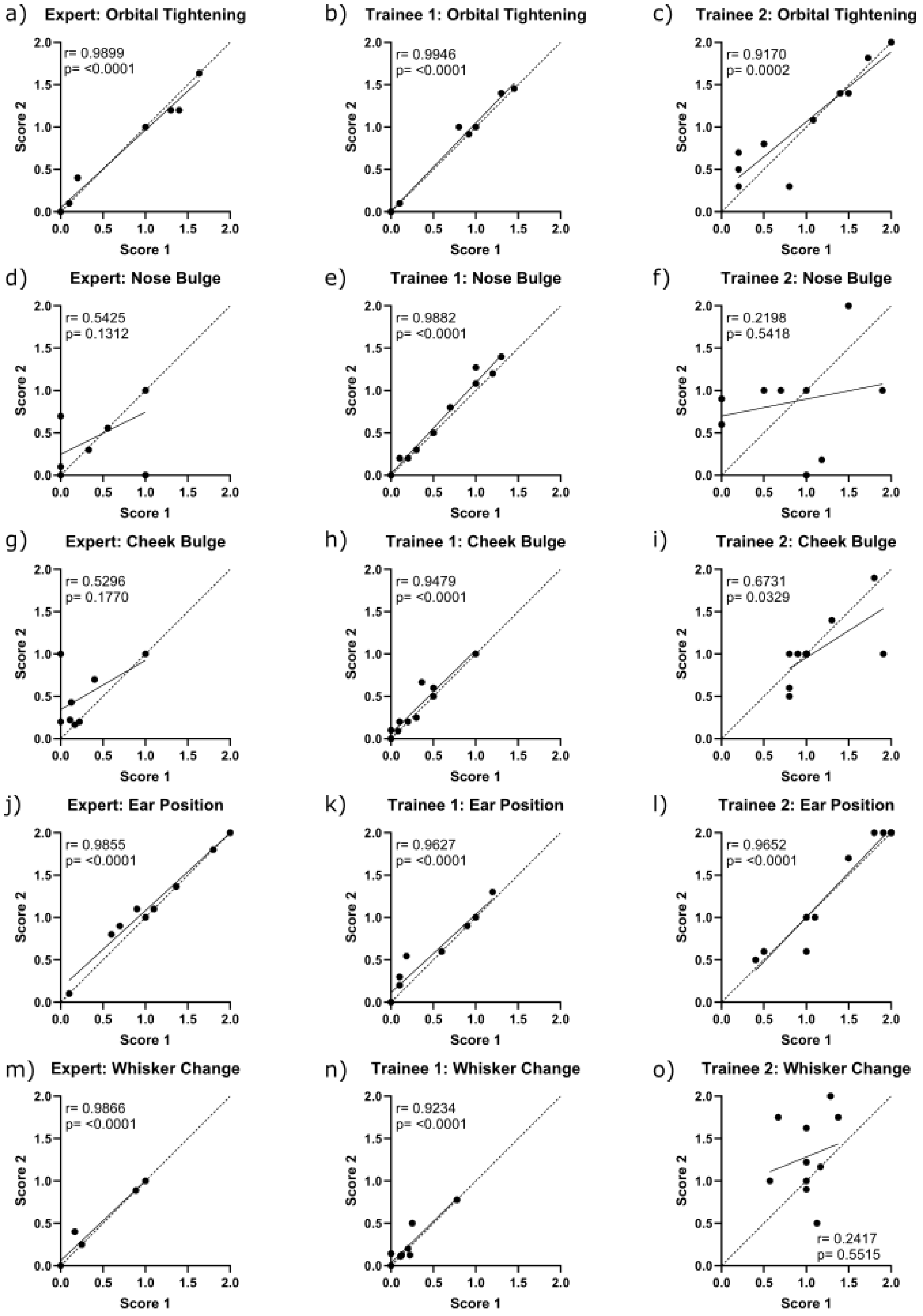
a) Expert: Orbital Tightening
r= 0.9899
p= <0.0001
b) Trainee 1: Orbital Tightening
r= 0.9946
p= <0.0001
c) Trainee 2: Orbital Tightening
r= 0.9170
p= 0.0002
d) Expert: Nose Bulge
r= 0.5425
p= 0.1312
e) Trainee 1: Nose Bulge
r= 0.9882
p= <0.0001
f) Trainee 2: Nose Bulge
r= 0.2198
p= 0.5418
g) Expert: Cheek Bulge
r= 0.5296
p= 0.1770
h) Trainee 1: Cheek Bulge
r= 0.9479
p= <0.0001
i) Trainee 2: Cheek Bulge
r= 0.6731
p= 0.0329
j) Expert: Ear Position
r= 0.9855
p= <0.0001
k) Trainee 1: Ear Position
r= 0.9627
p= <0.0001
l) Trainee 2: Ear Position
r= 0.9652
p= <0.0001
m) Expert: Whisker Change
r= 0.9866
p= <0.0001
n) Trainee 1: Whisker Change
r= 0.9234
p= <0.0001
o) Trainee 2: Whisker Change
r= 0.2417
p= 0.5515
Score 1
Score 2
0.0
0.5
1.0
1.5
2.0

Supplementary Figure 5. Intra-rater Pearson's correlations for individual grimace scale features. (a-c) orbital tightening, (d-f) nose bulge, (g-i) cheek bulge, (j-l) ear position, (m-o) whisker change. Orbital tightening and ear position show high intra-rater correlations, whereas nose bulge, cheek bulge and whisker change show lower correlations for some raters.

Experiment 1 (Fig 2) | Experiment 2 (Fig 3)

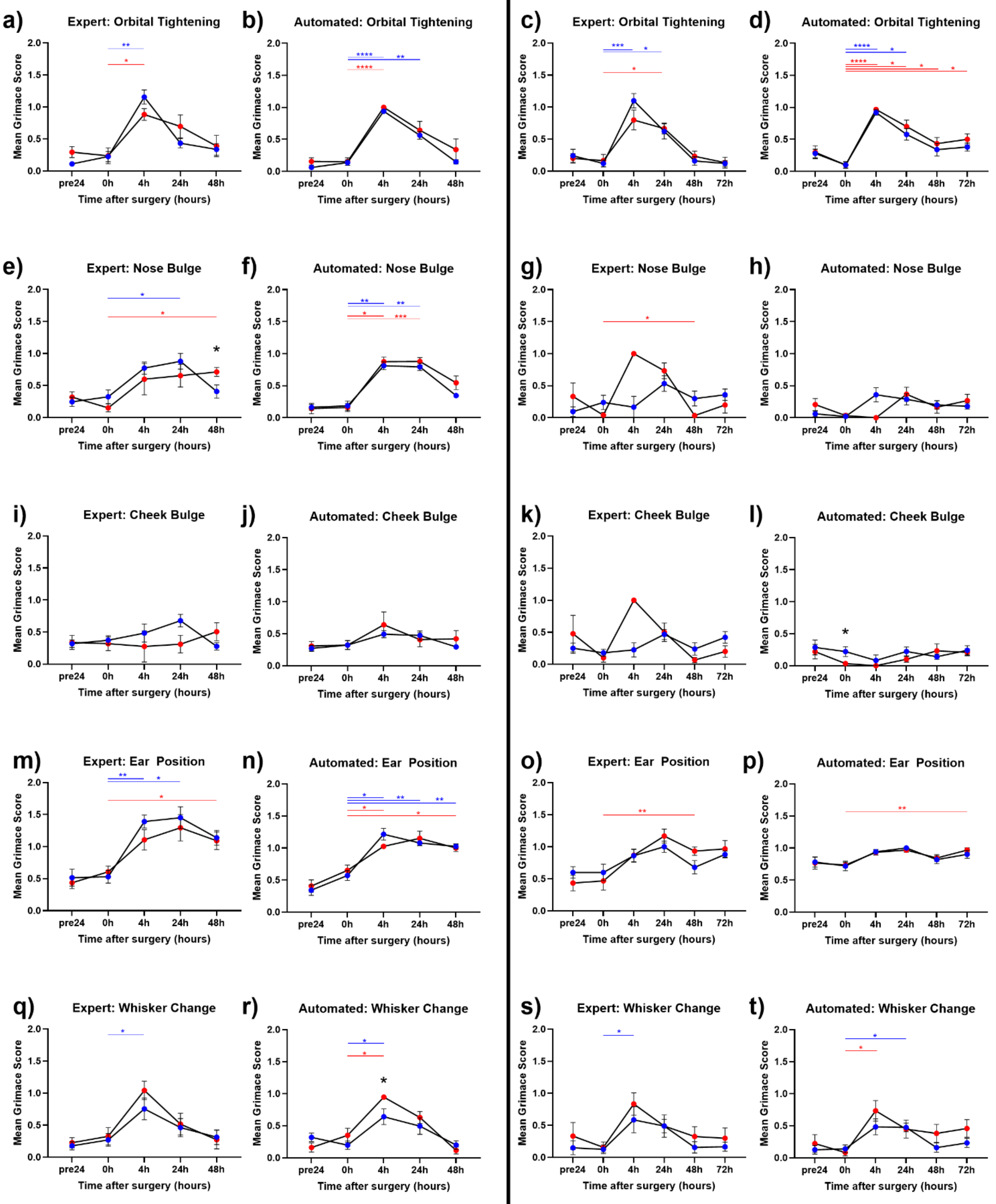

Supplementary Figure 6, Individual grimace scale features from expert and automated raters for Figures 2 and 3. (a) Orbital tightening: MGS scores from the expert rater show a significant main effect of time (F(3.309,41.36)=29.73, p=<0.0001) and a time x treatment interaction (F(4,50)=2.666, p=0.0429), but no effect on treatment. (b) Orbital tightening: MGS scores from the automated rater show a significant main effect of time (F (2.640, 41.57) = 58.70, p=<0.0001) and treatment (F (1, 63) = 4.076, p=0.0478) but no time x treatment interaction. (c) Orbital tightening: MGS scores from the expert rater show a significant main effect of time (F(3.054,42.14)=28.39, p=<0.0001) but no effect of treatment and no time x treatment interaction.(d) Orbital tightening: MGS scores from the automated rater show a significant main effect of time (F (3.543, 48.90) = 30.90, p=<0.0001) but no effect of treatment and no time x treatment interaction. (e) Nose Bulge: MGS scores from the expert rater show a significant main effect of time (F(2.212,26.54)=9.189, p=0.0007) but no effect of treatment and no time x treatment interaction. (f) Nose Bulge: MGS scores from the automated rater show a significant main effect of time (F(3.616,55.15)=52.65, p<0.0001) but no effect of treatment and no time x treatment interaction.(g) Nose Bulge: MGS scores from the expert rater show a significant main effect of time (F(3.293,39.52)=5.685, p=0.0019) and a time x treatment interaction (F(5,60)=3.189), p=0.0128, but no effect on treatment. (h) Nose Bulge: MGS scores from the automated rater show a significant main effect of time (F(3.435,41.22)=4.565, p=0.0055) but no effect of treatment and no time x treatment interaction. (i) Cheek Bulge: MGS scores from the expert rater show no significant main effects of time or treatment, and no time x treatment interaction. (j) Cheek Bulge: MGS scores from the automated rater show a significant main effect of time (F(3.504,51.68)=3.252, p=0.0234) but no effect of treatment, and no time x treatment interaction.(k) Cheek Bulge: MGS scores from the expert rater show a significant main effect of time (F(3.201,38.41)=4.084, p=0.0116) and a time x treatment interaction (F(5,60)=2.749, p=0.0266), but no effect on treatment. (l) Cheek Bulge: MGS scores from the automated rater show no significant main effects of time or treatment, and no time x treatment interaction. (m) Ear Position: MGS scores from the expert rater show a significant main effect of time (F(2.613,32.66)=19.21, p<0.0001) but no effect of treatment and no time x treatment interaction. (n) Ear Position: MGS scores from the automated rater show a significant main effect of time (F(2.989,37.36)=44.49, p<0.0001) but no effect of treatment and no time x treatment interaction. (o) Ear Position: MGS scores from the expert rater show a significant main effect of time (F(3.817,52.67)=10.31, p<0.0001) but no effect of treatment and no time x treatment interaction. (p) Ear Position: MGS scores from the automated rater show a significant main effect of time (F(2.314,31.94)=5.558, p=0.0063) but no effect of treatment and no time x treatment interaction. (q) Whisker Change: MGS scores from the expert rater show a significant main effect of time (F(3.308,39.70)=9.685, p<0.0001) but no effect of treatment and no time x treatment interaction. (r) Whisker Change: MGS scores from the automated rater show a significant main effect of time (F(2.805,33.66)=17.64, p<0.0001) but no effect of treatment and no time x treatment interaction. (s) Whisker Change: MGS scores from the expert rater show a significant main effect of time (F(2.481,40.69)=4.760, p=0.0093) but no effect of treatment and no time x treatment interaction. (t) Whisker Change: MGS scores from the automated rater show a significant main effect of time (F(2.955,40.19)=6.810, p=0.0009) but no effect of treatment and no time x treatment interaction. In panels a-t, asterisks represent significant Tukey's posthoc comparisons, smaller color-coded asterisks report drug vs time effects, larger black asterisks report between-group effects at a given timepoint.*=p<0.05, **=p<0.01, ***=p<0.001, ****=p<0.0001. Error bars represent SEM.

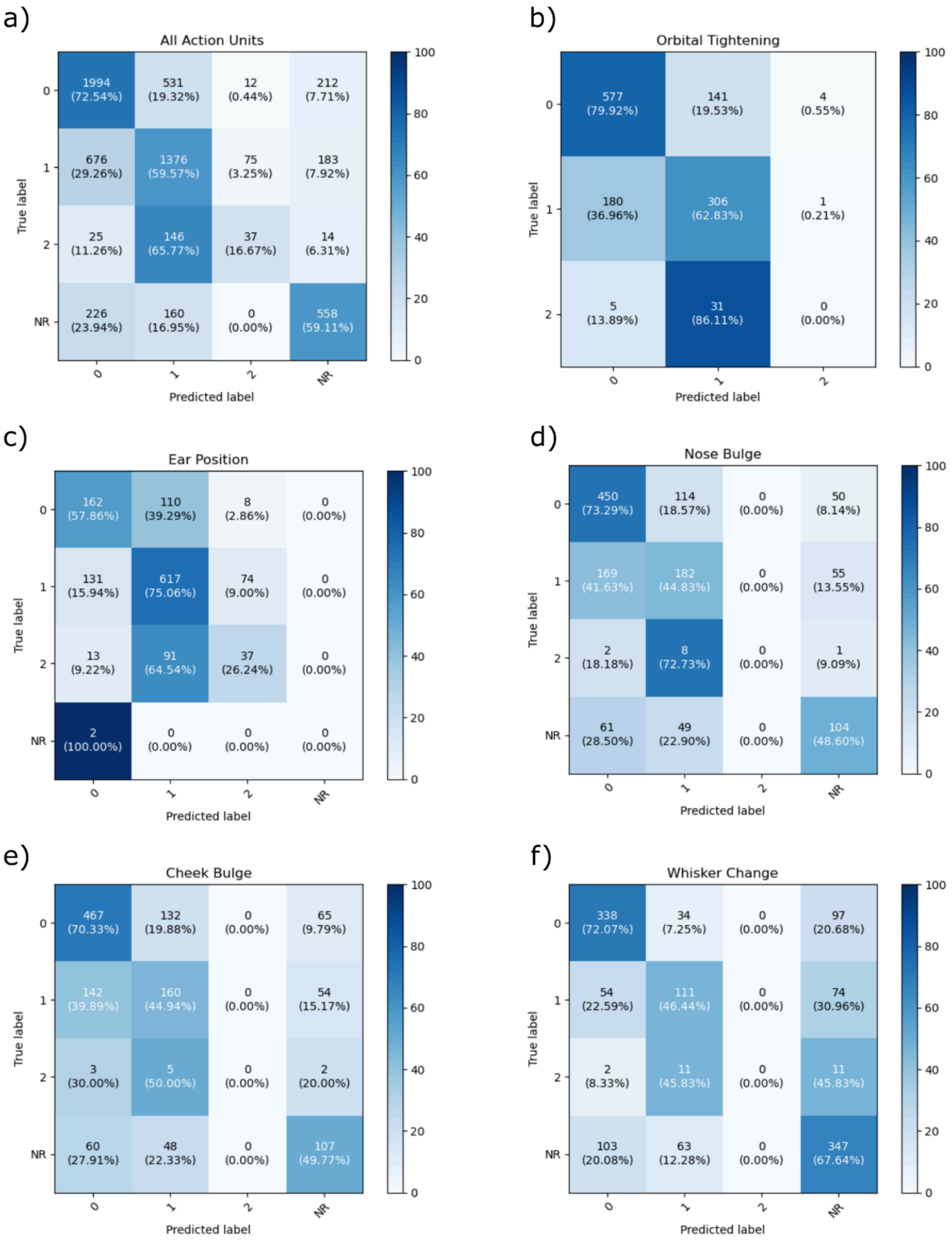

Supplementary Figure 7: Frame level comparisons between expert and automated scores. (a-f) MGS scores from the expert (true label) and automated scorer (predicted label) for all action units and each feature individually. Images that were not deemed scorable were marked as NR (not rateable).
